# Some Everyday Cases1Read before the meeting of the Pennsylvania State Veterinary Medical Association, September, 1899.

**Published:** 1900-01

**Authors:** Walker S. Phillips

**Affiliations:** Reading, Pa.


### REPORTS OF CASES.

### SOME EVERYDAY CASES.[1]

BY WALKER S. PHILLIPS, V.S.,

READING, PA.

VETERINARY science is progressing rapidly, and I am positive that the Pennsylvania Veterinary Medical Association has done much toward elevating and protecting its members.

When I recall the fall of 1860, the time I began to practise, remembering veterinary science as it was then, in its infancy, it is a great pleasure and satisfaction to me to see to-day how our profession has advanced, and how the farmers especially pay greater attention to their stock, have themselves much better judgment, and look for a veterinarian who can treat their stock scientifically. As I was perusing one of our valuable journals it occurred to me that the report of different cases which came under my observation might prove of some interest:

July 8th. The subject, an eight-year-old dapple-gray gelding, was observed two years ago to have a small tumor, situated on the lower part of the left flank, which had greatly increased in size. Upon examination I found it to be a melanotic tumor; the tumor had a base of about four inches. Dissecting it out and bringing the parts together with sutures could not be done. I removed with the knife, and had some trouble in checking the hemorrhage. The wound healed nicely without any sloughing of the parts, which is often the case with melanotic tumors.

[1] Read before the meeting of the Pennsylvania State Veterinary Medical Association, September, 1899.

A gray horse, somewhat aged, was used as a saddle-horse in this city for seven years by one family. Showed first symptoms as follows: After being brought in weakness across the loins; also on passing feces the passage would sometimes be obstructed and remain in the rectum. Upon examination per rectum I discovered a large, hard tumor on the right side of the rectum, in a straight line. Placed the hobbles upon the animal and cast him. Made an incision five inches in length on the right side of the urethra, and after further dissection removed a large melanotic tumor. The wound healed after some trouble with the usual discharge in such cases, considering the position of the wound. The animal was placed at work three weeks after the operation, and driven through a shower, after which symptoms of tetanus presented themselves, and as the animal was well up in years his recovery was not expected. Great interest was taken in the horse, as he was a faithful animal, and all efforts were made to save him, and he fully recovered.

On September 3d I was called to the country to see several calves about six weeks old. The owner stated that they breathed short and seemed to have swollen throats. Upon arriving at the stable found several animals affected with croup. Symptoms: Throat somewhat enlarged, breathing with difficulty, and a hoarse sound on coughing. Found the abdomen distended with gas in both cases, also high fever; no discharge from the bowels. Gave then to each animal two ounces of linseed-oil, applied a liniment to the throats, then also made a preparation composed of chlorate potash, powdered alum, spirits of nitre, mixed in honey, to be given three times daily. Before leaving the stable gave an injection to each animal. The owner, who has a milk route in this city, reported cases improved, and I cannot refrain from saying that in dangerous throat affections I find the above treatment has served me well in many a bad case, treating other symptoms as they present themselves, as indicated.

During the past two weeks have treated eight cases of spasmodic and flatulent colic, caused by feeding new oats. Medicines which I find most effective and give me satisfactory results are as follows: Chloric ether, tincture of opium, menthæ piperata; in flatulency, add bicarbonate soda and powdered zingiber.

Was called to see a bay horse, August 20th, eight years old, which had been coughing and breathed short. Was given a purgative. On arriving at the stable the proprietor said: "A queer case, a queer case." My diagnosis found the animal laboring with pleuro-pneumonia. The muscles in the lumbar region were congested and greatly swollen; also the muscles in the humeral region. Asked, was this horse driven? No. I was somewhat puzzled. Gave medicine for pleuro-pneumonia and applied a mustard sinapism to the chest. Still all symptoms of azoturia present. I again asked, was this horse out? The answer was, the owner had intended going out, but only got several squares and had to return to the stable. Second day, improvement in chest affection, but symptoms of azoturia worse. Partial loss of power of whole body. The animal dropped and laid quiet for an hour, then got up. Third day, same action. Then, in about a week or ten days, seemed well, and was driven as usual. The treatment in this case was a little difficult. Gave medicines first for the chest affection, then next dose I ventured on tonics, nux vomica, changing off as I saw fit.

August 14th, azoturia in a sorrel horse, six years old. Brought to my office with profuse perspiration, abdomen tympanitic, with considerable pain and pawing. Thought by the driver to be colic. Was affected in a short time with loss of power in the hind limbs; managed to get animal to stable eight squares away. Swelling of the muscles as usual. Horse, after standing five days, had recovered and was driven for two weeks, then was again attacked with the same disease. Upon looking into the case I found the animal had been fed more than the digestive organs could bear, and on resting a few days would be affected as above. The horse has been fed lighter since, and seems to be getting along nicely.

When I find cases as above in pain and tympanitis I treat those symptoms first. Apply stimulating liniments to the congested muscles after the pain has ceased, which usually lasts one hour, then I give my tonics and nerve medicine, very seldom purgatives. Have treated four cases in this city in the last four weeks.

